# Family and community pressure, obesity-related behaviours, and psychological wellbeing among female adolescents with overweight or obesity: a qualitative study

**DOI:** 10.3389/fpubh.2026.1945434

**Published:** 2026-10-07

**Authors:** Abeer Bahathig, Nasser S. Alqahtani

**Affiliations:** Department of Community Health, College of Applied Medical Sciences, Northern Border University, Arar, Saudi Arabia

**Keywords:** adolescent overweight and obesity, body image, family pressure, qualitative study, weight stigma

## Abstract

**Background:**

Adolescent obesity has physical, psychological and social consequences. Family and community influences can be important in determining the eating behaviour, body image and emotional health of adolescents. Few qualitative studies have investigated female adolescents’ own perception of family and community related pressure around their weight.

**Methods:**

This qualitative exploratory study used semi-structured interviews to investigate these experiences among 30 female adolescents aged 12–19 years (mean 16.3 years) with overweight or obesity. Weight status was classified against the WHO 2007 BMI-for-age reference. The study covered eating behaviour, criticism, emotional impact, and perceived approaches to managing obesity. Data were analysed using inductive thematic analysis.

**Results:**

Seventeen participants (57%) met WHO criteria for obesity and 13 (43%) for overweight. Four themes emerged: (1) a conflicted relationship with food, characterised by dieting, restrained eating, and a cycle of restriction followed by overeating (*n* = 16); (2) experiences of criticism from parents, family members and the wider community (*n* = 20), most commonly around appearance and health; (3) psychological and life-choice implications, including self-consciousness and embarrassment (*n* = 25), low self-esteem, social withdrawal, and concerns about clothing, autonomy and future marriage (*n* = 22); and (4) motivation and perceived strategies for obesity management, including healthy eating, dieting, exercise, and medical interventions. Negative comments were mostly reported as unwelcome, whereas positive and health-related comments were reported as motivating.

**Conclusion:**

Pressure from family and community is a key component in how female adolescents with overweight or obesity perceive their weight, influencing behaviour, emotions, and social functioning. Obesity interventions should incorporate mental health, stigma reduction, and support for constructive family and community communication, alongside weight management.

## 1 Introduction

Obesity in adolescents is a major health challenge that has severe physical, psychological, and sociological implications (1). In the last 10 years, many extensive research studies have indicated a considerable increase in overweight and obesity in children and adolescents around the world (2, 3). With adolescent obesity there are not only health risks but also psychological consequences, such as emotional distress, stigma, and poor body image, which can result in a lower quality of life. Adolescence is an identity formation stage in which young people become highly sensitive to social evaluations; weight experiences may therefore be especially influential on mental wellbeing and behaviour (4).

It is widely accepted that family plays an important role in adolescent eating habits, physical activity, and attitudes towards body weight. The family environment shapes food practises, nutrition, and body image and weight concerns (5, 6). While constructive family participation might promote positive behaviour, weight-based teasing or criticism, comparisons, and constant commentary regarding appearance or wellbeing can result in stigma and emotional suffering (7, 8). There is evidence that negative interactions about weight are related to body dissatisfaction, low self-esteem, emotional disturbance, and problematic eating behaviours (9, 10). In contrast, supportive and respectful communication appears more beneficial for adolescent wellbeing (11, 12).

Though some researchers have studied the relationship between obesity, parental behaviour, and psychological outcomes, few have investigated how adolescents themselves make sense of obesity-related pressures within families and communities (13, 14). Emerging qualitative evidence from Saudi Arabia has examined how adolescents perceive various determinants of obesity, including diet, the health-care system, and stigmatisation (15). However, there is no qualitative investigation into the interactional processes and pressures around weight among female adolescents within families and communities. This study therefore sought to investigate the experience of family and community pressure related to weight among female adolescents with overweight or obesity, and its effects on their eating behaviours, body image, and mental wellbeing.

## 2 Materials and methods

### 2.1 Study design

This exploratory qualitative study examined the experiences of female adolescents facing family and community pressure related to their weight, and the effects of this on their eating habits and psychological wellbeing. A qualitative exploratory design (16) was used to focus on how adolescents interpreted criticism, support, and weight-related pressure within everyday family and social life (8, 17). Reflexive thematic analysis (18) was used to identify recurrent patterns across participants’ narratives. Qualitative inquiry was considered appropriate because weight and weight-related communication are often experienced as sensitive and emotionally charged (12, 17). Reporting follows the Consolidated Criteria for Reporting Qualitative Research (COREQ) (19); the completed checklist is provided as Supplementary material.

### 2.2 Study setting

The research was conducted in intermediate and secondary schools located in Riyadh and Arar, Saudi Arabia. Conducting the research in schools provided access to female adolescents who had experienced family and societal pressure concerning their weight, in a naturalistic setting (20).

### 2.3 Participants

Participants were female adolescents aged 12–19 years with overweight or obesity. The study focused on adolescence because this stage marks a critical point in development at which body image and family and social evaluation become increasingly salient. A total of 30 adolescents comprised the final sample.

Adolescents were eligible if they were female, aged 12–19 years, had a body mass index (BMI) above the age- and sex-specific threshold for overweight, were able to describe their experiences verbally, and had written consent from a parent or guardian. Adolescents with severe cognitive impairment, or with medical or psychiatric conditions that would have prevented participation, were excluded. Height and weight were measured by the research team to the nearest centimetre and kilogramme at the time of recruitment. BMI was calculated and classified *post hoc* against the WHO 2007 BMI-for-age reference for girls aged 5–19 years (21), using age in completed years, with overweight defined as > +1 SD and obesity as > +2 SD. On this basis 17 participants (56.7%) met criteria for obesity and 13 (43.3%) for overweight; weight status is reported in full in Table 1. Because the sample spans both categories, the population is described throughout as female adolescents with overweight or obesity.

**Table 1:** Participant characteristics (*N* = 30).

Characteristic	Value
Age, years—mean (SD)	16.3 (1.9)
Age, years—range	12–19
Age group, *n* (%)—12–14 years	6 (20.0)
Age group, *n* (%)—15–17 years	16 (53.3)
Age group, *n* (%)—18–19 years	8 (26.7)
Height, cm—mean (SD)	157.1 (8.2)
Weight, kg—mean (SD)	76.4 (11.9)
BMI, kg/m^2^—mean (SD)	31.1 (5.0)
BMI, kg/m^2^—range	24.8–43.1
BMI-for-age *z*-score—mean (SD)	2.27 (0.73)
BMI-for-age *z*-score—range	1.20–3.66
Weight status, *n* (%)—obesity (*z* > +2 SD)	17 (56.7)
Weight status, *n* (%)—overweight (+1 SD < *z* ≤ +2 SD)	13 (43.3)
Interview duration, minutes—range	30–45

BMI-for-age *z*-scores were calculated against the WHO 2007 growth reference for girls aged 5–19 years (21), using age in completed years. Overweight was defined as *z* > +1 SD and obesity as *z* > +2 SD.

### 2.4 Sampling strategy

Recruitment began with school principals across the participating cities, who supported BMI screening of female students. Students identified at screening as having a high BMI for their age were considered for invitation. A purposive, criterion-based sampling strategy (22) was then used to invite these students to interview, in order to obtain information-rich accounts of family and community pressure, eating behaviour, and emotional responses. Thirty-one eligible adolescents were invited to participate. One declined, and the final sample comprised 30 female adolescents. Participants were recruited from school settings in Saudi Arabia, allowing exploration of adolescents’ weight-related experiences within their educational and family contexts.

### 2.5 Sample size

The final sample comprised 30 female adolescents aged 12–19 years (mean 16.3 years, SD 1.9). Sample size was guided by information power rather than a predetermined target (23): the study aim was narrow, participants were selected against specific criteria, and the interviews yielded dense accounts on the topics of interest. Data collection and analysis proceeded in parallel so that emerging codes could inform later interviews. Recruitment stopped when the final interviews generated no new codes relevant to the study aim.

### 2.6 Data collection tool

Data were gathered using a semi-structured interview guide designed to examine adolescents’ experience of their weight and of family and community pressure. The guide was developed from the literature on adolescent obesity, family communication and weight-related pressure. Interviews covered eating behaviour, family and social conversations about weight, criticism, feelings of being compared, emotional responses, and coping or support needs. Open-ended questions and follow-up prompts were used to encourage detailed discussion. Interviews were conducted in Arabic. Recordings were transcribed verbatim in Arabic and coded in Arabic. Quotations selected for reporting were translated into English by the research team.

### 2.7 Interview procedure

Individual, semi-structured interviews were conducted by the first author (AB), an academic in community health with experience in qualitative interviewing and in discussing sensitive topics with adolescents. Each participant took part in one interview only; no follow-up interviews were conducted. Interviews were held privately on school premises, without parents or school staff present, lasted 30–45 min, and were audio recorded with participants’ consent. Two female master’s students assisted with recruitment and with scheduling interviews; they did not conduct interviews and took no part in coding or analysis.

Participants were assured that all information they provided would be kept confidential and anonymous, and that participation was voluntary.

### 2.8 Data analysis

Interview recordings were transcribed verbatim, and the data were analysed using inductive thematic analysis (18), with coding and data organisation performed in Microsoft Excel (24). The first author read the transcripts and field notes repeatedly to become familiar with the data. Initial codes were generated inductively, focusing on participants’ accounts of family pressure, eating behaviour, emotional responses, body image, and coping strategies. Similar codes were grouped into broader categories, from which themes and subthemes were developed through an iterative process of constant comparison across the dataset. Themes were reviewed and refined to ensure they represented participants’ accounts and remained grounded in the data. Representative verbatim quotations were selected to illustrate each theme.

The number of participants contributing to each code was recorded, and frequency terms are used consistently throughout the results: “a few” denotes 2–4 participants, “some” 5–9, “many” 10–19, “most” 20–29, and “all” 30. Participant counts are given in parentheses where each subtheme is introduced. One further code (family weight-related tension, *n* = 6) overlapped substantially with criticism from parents and other family members and was not retained as a separate subtheme.

### 2.9 Trustworthiness and rigour

Trustworthiness was addressed against the criteria of credibility, dependability, confirmability, and transferability (25). Credibility was supported by repeated engagement with the data, repeated reading of transcripts, and the use of verbatim quotations to keep the findings anchored to participants’ accounts. Dependability was supported by applying the same documented analytic procedure throughout coding and theme development. Confirmability was supported by grounding interpretations in participants’ narratives and by maintaining an audit trail of coding decisions and theme development. The second author independently reviewed the coding frame and the theme structure against the coded data; this was a review of the analytic framework rather than independent double coding of transcripts.

### 2.10 Researcher reflexivity

All interviews were conducted by the first author (AB), a female academic in community health with prior experience in qualitative interviewing. She had no prior relationship with any participant. Participants were told that the interviewer was a female researcher from the university with an interest in adolescent health and family relationships; no further personal information was shared.

The first author approached the topic from a public health background, with a prior view that weight-related comments within families are frequently experienced as harmful even when they are intended supportively. This position shaped the interview guide, which asks directly about criticism and comparison, and may have made accounts of distress more accessible in the interviews than accounts of neutral or welcome family involvement. It was kept in view during analysis by writing memos after each interview and by returning to transcripts where coding appeared to run ahead of what participants had actually said. Where participants described family comments as helpful, these accounts were coded and retained rather than treated as exceptions, and they form the basis of Theme 4.

The first author is an adult university researcher and the participants were schoolgirls. This authority difference may have limited what some participants were willing to disclose, particularly among the youngest, and three participants in the 12–14 year group gave notably brief responses. Interviews were held privately, without parents or school staff present, to reduce this constraint.

### 2.11 Ethical considerations

Ethical approval was obtained from the Ethics Committee of Northern Border University (approval number HAP-09-43); approved prior to data collection. In addition to written informed consent from parents or guardians, written assent was obtained from the adolescent participants. Participants were informed of the purpose of the study, that participation was voluntary, and that they could withdraw at any time without consequence.

Data were anonymised and kept confidential. Transcripts were coded and no other identifying information was attached; data were kept secure and accessible only to the research team. Interviews were conducted carefully and sensitively in relation to weight, stigma, and body image.

## 3 Results

The interviews generated four main themes: (1) relationship with food, (2) experiences of criticism, (3) psychological and life-choice implications, and (4) motivation and perceived approaches to weight management. Together these themes show that weight was experienced not only as a physical matter but as a social and emotional one, embedded in family and community relationships. Themes, subthemes and participant counts are shown in Table 2.

**Table 2:** Themes, subthemes, participant counts, and illustrative quotations.

Theme	Subtheme	*n*/30	Illustrative quotation
1. Relationship with food	Fast food	5	“It is difficult to stop eating fast food because it tastes delicious” (P6).
1. Relationship with food	Healthy eating	6	–
1. Relationship with food	Dieting	15	“I regained the weight” (P24).
1. Relationship with food	Restraint paradox	16	“Sometimes I restrain myself from eating for long periods, but then I end up eating chips and sweets” (P7).
2. Experiences of criticism	Source: parents	9	“They put pressure on me to lose weight” (P10).
2. Experiences of criticism	Source: extended family	14	“Why are you so fat? You need to lose some weight” (P14).
2. Experiences of criticism	Source: wider community	10	“They always linked obesity to marriage opportunities, saying that no one would marry me the way I looked” (P14).
2. Experiences of criticism	Nature: appearance	15	“If you were thinner…” (P4).
2. Experiences of criticism	Nature: bullying or humiliation	10	“My uncle would always upset me during family gatherings, and I would often cry … each comment reduced my self-confidence” (P14).
2. Experiences of criticism	Nature: health concerns	11	“… such as diabetes and heart problems” (P16).
2. Experiences of criticism	Nature: social comparison	12	“Marriage is for people with slim bodies, and obesity makes you unattractive” (P18).
3. Psychological and life-choice implications	Self-consciousness and embarrassment	25	“My self-confidence decreased every time I looked in the mirror and remembered their remarks” (P11).
3. Psychological and life-choice implications	Social withdrawal	9	“I completely avoid being photographed because I do not consider myself beautiful” (P3).
3. Psychological and life-choice implications	Self-judgement	4	—
3. Psychological and life-choice implications	Marriage prospects	22	“Marriage is for people with slim bodies, and obesity makes you unattractive” (P18).
3. Psychological and life-choice implications	Clothing	5	“Whenever I buy clothes, I hear many comments that upset me … ‘If you were thinner…’” (P4).
3. Psychological and life-choice implications	Reduced autonomy	2	“This makes me let them choose my clothes, so I do not hear constant criticism” (P4).
4. Motivation and perceived approaches	Motivation from health-framed comments	7	“My family continuously supported me and stood by my side until I was able to reach a healthier and more suitable weight” (P10).
4. Motivation and perceived approaches	Physical activity	10	–
4. Motivation and perceived approaches	Medical or surgical intervention	7	–

Counts are the number of participants (of 30) whose accounts contributed to each subtheme. Dieting and healthy eating appear under both Theme 1 and Theme 4; the counts refer to the same codes and are not additive.

### 3.1 Participant characteristics

Thirty female adolescents aged 12–19 years participated (mean 16.3 years, SD 1.9). Seventeen (56.7%) met WHO 2007 criteria for obesity and 13 (43.3%) for overweight. Mean BMI was 31.1 kg/m^2^ (SD 5.0, range 24.8–43.1) and mean BMI-for-age *z*-score was 2.27 (SD 0.73, range 1.20–3.66). Participant characteristics are presented in Table 1.

Three participants in the 12–14 year group (P1, P2, P27) gave notably brief responses and declined to expand when asked about criticism; their interviews yielded 5, 4 and 0 coded segments respectively, against a sample mean of 8.3. One of these (P27) reported no negative experiences related to her weight and was the only participant for whom no codes were generated. Older adolescents generally described more detailed experiences of criticism, self-esteem and coping than younger ones.

### 3.2 Overview of themes

Four domains were identified. The first was participants’ relationship with food, covering fast food, healthy eating, dieting and the restraint paradox. The second concerned experiences of criticism, including its sources and its content. The third covered the psychological and life-choice consequences of these experiences, including self-consciousness, lowered self-esteem, social withdrawal, clothing, and expectations about marriage. The fourth covered motivation and perceived approaches to weight management through healthy eating, dieting, physical activity and medical intervention. Table 2 presents themes, subthemes, participant counts and illustrative quotations.

### 3.3 Theme 1: relationship with food

Participants described complex and often conflicted relationships with food. Four subthemes were identified: fast food, healthy eating, dieting, and the restraint paradox. Many participants acknowledged that healthy eating mattered while also describing difficulty giving up foods they found enjoyable or comforting.

#### 3.3.1 Fast food as an obstacle

Five participants identified fast food as an obstacle to controlling their weight and described difficulty resisting foods they found pleasurable or comforting. As one explained, “It is difficult to stop eating fast food because it tastes delicious” (P6). These accounts point to a link between eating and pleasure that goes beyond nutrition.

#### 3.3.2 Healthy eating as an intentional strategy

Six participants described healthy eating as their preferred approach to weight management, framing it as improving what they ate rather than restricting how much.

#### 3.3.3 Dieting

Dieting was the most commonly reported weight-loss strategy (*n* = 15). Participants described repeated attempts and repeated failure. One stated simply, “I regained the weight” (P24), reflecting the frustration of cycles of loss and regain.

#### 3.3.4 The restraint paradox

Sixteen participants described periods of rigid food restriction followed by rebound eating. As one put it, “Sometimes I restrain myself from eating for long periods, but then I end up eating chips and sweets” (P7). Another reported that restricting food did “not change anything” (P4). These accounts suggest that restriction more often ended in frustration and rebound than in sustained weight control.

Taken together, these accounts describe conflicted relationships with food marked by repeated dieting, restraint, and difficulty sustaining behaviour change.

### 3.4 Theme 2: experiences of criticism

Criticism was among the most emotionally charged topics raised. Twenty participants described experiences of weight-related criticism. It came from parents (*n* = 9), extended family members (*n* = 14), and the wider social environment (*n* = 10), indicating that weight-related judgement occurred across several interpersonal contexts rather than within the family alone.

#### 3.4.1 Appearance-based criticism

Comments about appearance were the most frequent form of criticism (*n* = 15). Participants described feeling judged on how they looked, which heightened self-consciousness and fear of others’ opinions.

#### 3.4.2 Bullying and humiliating remarks

Ten participants recounted criticism that crossed into bullying or humiliation. One explained:

*“My uncle would always upset me during family gatherings, and I would often cry. He would say things like, ‘Why are you so fat? You need to lose some weight.’ … I was particularly affected by my uncle’s words; I would always remember them and continue crying, and each comment reduced my self-confidence” (P14).*

This account illustrates how repeated criticism from family members was experienced as emotionally damaging and connected to lowered self-worth.

#### 3.4.3 Health concerns as criticism

Eleven participants described comments framed as concern for their physical health. Reminders about weight, or warnings about future health risks, were still often experienced as coercive, depending on how they were delivered.

#### 3.4.4 Social comparison

Twelve participants described being compared with socially preferred body types or with people seen as slimmer. These comparisons reinforced feelings of insecurity.

Across its forms—appearance-based, comparative, bullying, and health-framed—criticism was described as common and emotionally upsetting. Notably, criticism and support were not always separable: several participants described the same family members as both pressuring and supporting them, and one account of family support (P10) also describes repeated instruction to lose weight.

### 3.5 Theme 3: psychological and life-choice implications

Interviews highlighted psychological and social consequences of weight-related criticism, including self-consciousness, low self-esteem, social withdrawal, and concerns about future life prospects.

#### 3.5.1 Psychological implications

Self-consciousness and embarrassment about weight were the most frequently coded experience in the dataset (*n* = 25). Participants described shame and emotional distress in response to repeated negative comments, and some described feeling “suffocated” by remarks about their weight (P16, P21, P24). Nine participants described withdrawing from social situations. As one explained, “I completely avoid being photographed because I do not consider myself beautiful” (P3), and another said, “My self-confidence decreased every time I looked in the mirror and remembered their remarks” (P11). A few participants (*n* = 4) also turned criticism inward, interpreting unsuccessful weight-loss attempts as personal failure.

#### 3.5.2 Life-choice implications

Experiences of weight and criticism also shaped everyday decisions and expectations about the future. Concern that weight would reduce future marriage prospects was described by most participants (*n* = 22). As one put it, “Marriage is for people with slim bodies, and obesity makes you unattractive” (P18). Criticism also affected clothing choices (*n* = 5) and personal autonomy (*n* = 2). One participant explained:

*“Whenever I buy clothes, I hear many comments that upset me, such as, ‘This does not suit you,’ ‘It’s not nice on you,’ or ‘If you were thinner…’ This makes me let them choose my clothes, so I do not hear constant criticism” (P4)*.

Some adolescents avoided particular clothing styles, or allowed others to make appearance-related decisions on their behalf, in order to reduce criticism. Criticism therefore affected emotional wellbeing, self-presentation, and outlook on the future.

### 3.6 Theme 4: motivation and perceived approaches to weight management

Although criticism was generally experienced negatively, some participants described weight-related comments as motivating when they were framed around health rather than appearance.

#### 3.6.1 Motivation

Seven participants reported that comments about their weight encouraged healthier behaviour, particularly where family members emphasised preventing future health problems such as “diabetes and heart problems” (P16). Supportive experiences were described more often in families where other members also lived with overweight or obesity.

*“My family continuously supported me and stood by my side until I was able to reach a healthier and more suitable weight. They would always tell me that I need to reduce my weight so that it does not negatively affect my health” (P10)*.

This account also shows the ambivalence noted above: the same family communication is described as supportive and as a repeated instruction to lose weight. Where communication about health was experienced as supportive rather than critical or shaming, adolescents appeared more open to family involvement.

#### 3.6.2 Perceived approaches to management

Participants identified a range of weight-management strategies: dieting (*n* = 15), physical activity (*n* = 10), healthy eating (*n* = 6), and medical or surgical treatment (*n* = 7). Medication and gastric surgery were mentioned by a minority, reflecting a view that weight sometimes requires clinical as well as behavioural management. Across these strategies, supportive communication was consistently described as more helpful than criticism in motivating behaviour change.

### 3.7 Summary of results

Participants described their weight as a social and emotional experience as much as a physical one, shaped by eating behaviour, criticism, self-perception and expectations about the future. Conflicted relationships with food and exposure to weight-related criticism were common, with psychological and social consequences. Some participants, however, described supportive and health-focused family involvement as motivating, and identified healthier eating, physical activity and medical support as possible approaches to weight management.

The interrelationships between family pressure, emotional responses, eating behaviour, coping strategies, and support needs are summarised in Figure 1.

**Figure 1:**
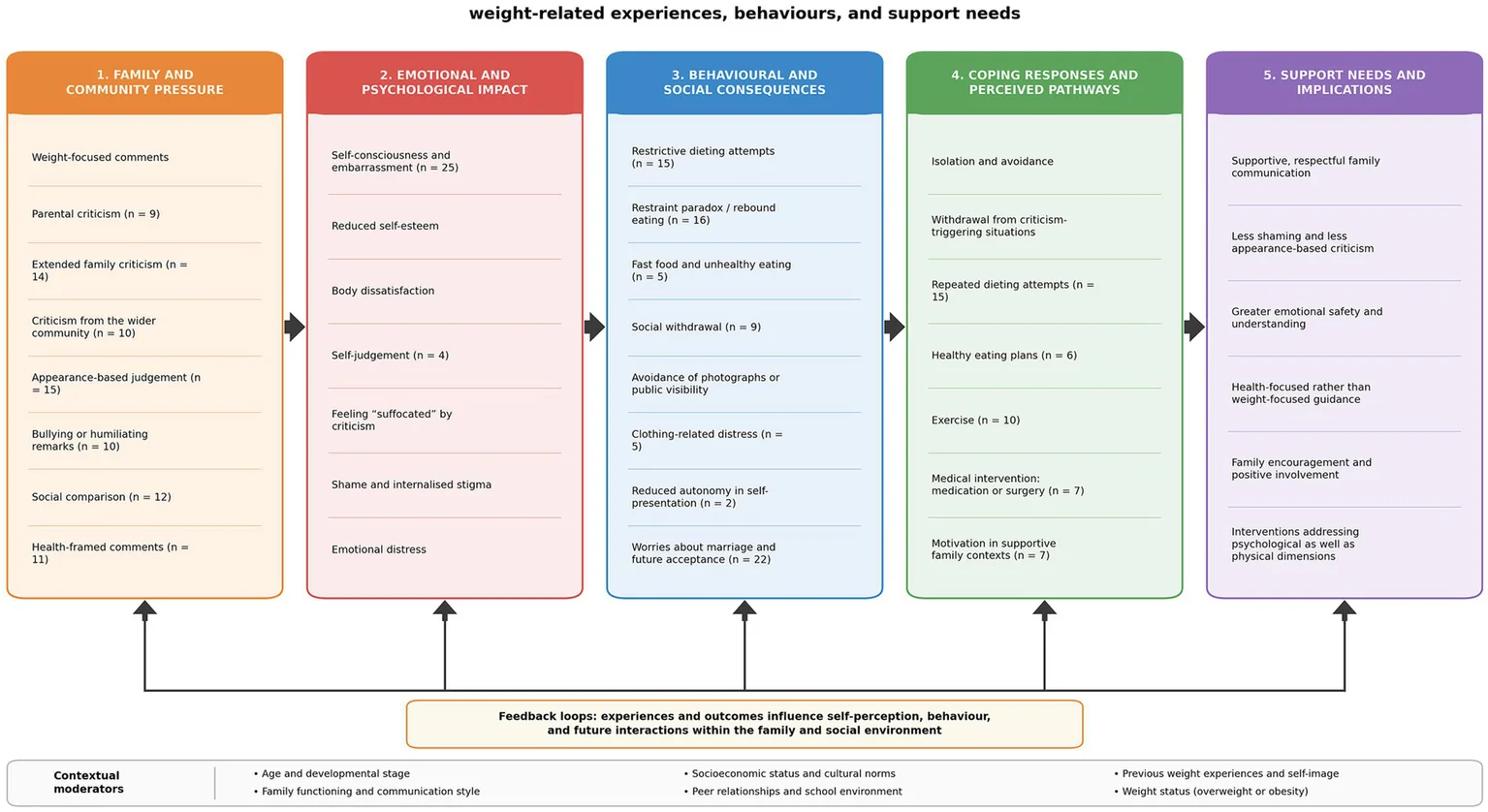
Conceptual framework of the findings. This framework illustrates the interrelationships identified between family and community pressure, weight-related criticism, and adolescents’ psychological wellbeing, eating behaviour, social functioning, and perceived support needs, as described by participants.

## 4 Discussion

### 4.1 Principal findings

This study explored female adolescents’ experiences of family and community pressure related to their weight, and its influence on eating behaviour, body image, and psychological wellbeing. Four themes were identified: (i) a conflicted relationship with food; (ii) experiences of criticism from parents, family, and the wider community; (iii) psychological and life-choice implications of weight-related stigma; and (iv) motivation and perceived approaches to weight management. These findings indicate that weight among female adolescents is experienced not only as a physical condition but as a relational and emotional one, shaped by family and community interactions (26).

The findings reinforce the view that adolescent overweight and obesity should be understood as a biopsychosocial condition, in which family communication, social expectations, and emotional wellbeing interact with health behaviour. Rather than treating weight solely as a matter of individual lifestyle choice, the findings suggest that supportive family and community environments may support healthier behaviour while limiting the harmful effects of weight stigma (27).

### 4.2 Theme 1: a conflicted relationship with food

Participants described cycles of dietary restriction and overeating, and recognised that food played both emotional and physical roles in their lives. This is consistent with findings identifying food as a source of comfort as well as nutrition (6), and builds on research showing that the parenting environment and parent–adolescent connectedness influence eating behaviour and impulse control (13, 14). This study adds adolescents’ own perspective that repeated failed restriction became a source of frustration in itself. Participants did not simply struggle to diet; they also recognised that dieting alone was insufficient. This is consistent with the view that weight management cannot be separated from its emotional and relational context (8, 17).

### 4.3 Theme 2: criticism from family and community

Criticism was described by two thirds of participants (*n* = 20) and was the most emotionally charged topic in the interviews. Encouragement and criticism frequently came from the same family members, and participants often described receiving support alongside repeated comments about their weight (7, 8). Support and criticism therefore coexisted within the same relationships: comments intended to encourage healthier behaviour were still experienced as critical, particularly when repeated or comparative. This gap between intent and perceived impact has been reported previously, including evidence that positive and negative parental weight comments frequently coexist within the same family relationships (28). The present findings also suggest that the social context in which criticism occurs—for example in front of extended family rather than in private—may further shape how it is experienced, an area that would benefit from targeted investigation. Consistent with previous work, criticism did not generally motivate positive behaviour change; instead it was associated with reduced self-esteem and reinforced feelings of inadequacy (11).

### 4.4 Theme 3: psychological and life-choice implications

Participants’ accounts reflect adolescence as a particularly sensitive period for body image development. Self-consciousness and embarrassment were the most frequently coded experience in the dataset (*n* = 25), and repeated weight-related criticism was described as damaging to self-esteem. Nine participants described withdrawing from social situations, consistent with previous work linking criticism to social withdrawal (1, 11). Several avoided being photographed and worried about being judged, which accords with studies linking weight-related comments to body dissatisfaction and emotional distress (7, 10).

A further finding was the strength of the perceived link between body weight and marriage prospects, described by most participants (*n* = 22). Participants explained that being thin was necessary for social acceptance and for being considered suitable for marriage. Previous research has described internalised weight stigma (9); these findings suggest that, in this setting, the perceived link between body weight and marriageability was a specific and prominent form of it, shaping both self-perception and expectations about the future.

### 4.5 Theme 4: motivation and perceived approaches to weight management

Participants distinguished clearly between supportive and critical family communication and its effect on their motivation. Comments focused on health were experienced as more encouraging than comments about appearance. Where family members had themselves lived with overweight or obesity, such conversations were more often read as genuine concern rather than criticism or blame. Previous research has reported similar findings, showing that adolescents respond better to respectful discussions about health than to comments about appearance or shame. Studies also suggest that both adolescents and parents prefer indirect and supportive conversations about weight rather than direct confrontation (29). Many participants described coping by avoiding social activities, avoiding photographs, or changing the way they dressed. These responses reflected a loss of confidence and reduced independence rather than an attempt to manage their weight. When discussing ways to address their weight, participants most often referred to healthy eating, dieting, regular exercise and, for some, medical or surgical treatment. Their accounts suggest that they viewed weight as a complex issue requiring behavioural change, emotional support and, in some cases, clinical care, rather than self-control alone (1).

### 4.6 Physical activity: recommendations and the local context

Exercise was named as a route to weight management by 10 participants. This study did not measure physical activity, and no participant was asked about access to sport or about attitudes towards girls taking part in it. What follows is therefore context for interpreting those accounts rather than a finding of this study.

WHO recommends that children and adolescents aged 5–17 years undertake an average of at least 60 min per day of moderate- to vigorous-intensity, mostly aerobic, physical activity across the week; that vigorous-intensity activity and activities strengthening muscle and bone be incorporated on at least 3 days per week; and that sedentary time, particularly recreational screen time, be limited (30). Meeting these recommendations is a general requirement of adolescent health rather than a treatment for excess weight, and the distinction matters for this sample. Where activity is presented to girls specifically as a corrective for body size, it is offered in the same register as the appearance-focused comments that participants described as discouraging, and it risks being received as one more form of weight-related pressure. Framing activity as a norm for all adolescents, linked to enjoyment, competence and health, is more consistent with the kind of communication participants described as motivating.

Attainment of these recommendations among Saudi adolescent girls is low. Among 399 girls aged 13–14 years in Arar, one of the two cities in the present study, 92.7% did not meet the recommendation for daily moderate-to-vigorous activity, and mean sedentary time exceeded 6 h on weekdays and seven and a half hours at weekends (31). Nationally, girls are consistently less active and more sedentary than boys (32).

Part of this gap is structural rather than motivational. Physical education was not available to girls in Saudi public schools until 2017, when the Ministry of Education approved its introduction, to be implemented gradually and in accordance with regulatory requirements; provision has since depended on the availability of suitable facilities and qualified female instructors, and the expansion of sport and physical activity for women and girls forms part of the Vision 2030 quality of life agenda (33). The participants in this study were schooled across that transition. Opportunity to be active has therefore not been equally available to girls and boys in this setting, and interventions addressing individual motivation alone are unlikely to close the gap. A school-based intervention combining nutrition, physical activity and body image acceptance among adolescent girls in Arar improved knowledge, attitudes and practise, indicating that such approaches are feasible locally (34).

Whether the girls in this study experienced sport as available to them, as socially acceptable, or as something directed mainly at those with higher weight, was not asked and cannot be answered from these data. It is a question worth putting to adolescents directly in future work, because the answer bears on whether promoting physical activity reduces weight-related stigma or reinforces it.

### 4.7 What this study adds

Much of the existing literature on family communication and adolescent weight-related distress comes from North American and European contexts (7, 12–14, 28). Recent qualitative studies from Saudi Arabia have explored how adolescents perceive the factors contributing to obesity, including dietary habits, health system influences, and weight-related stigma (15), but have not addressed the interpersonal, family- and community-level dynamics of weight-related pressure among female adolescents. These findings add to existing knowledge in three respects. Participants described receiving both criticism and support from the same people, emphasised the role of marriage-related stigma, and explained that pressure often came from the wider community rather than from family alone. The results indicate that interventions for adolescent overweight and obesity should extend beyond dietary and physical activity counselling to include psychosocial support and strategies to reduce weight stigma across the family. Education for parents and carers on supportive communication that avoids weight-related shame should be incorporated into school and community-based programmes, since in this sample criticism more commonly triggered distress than behaviour change.

### 4.8 Strengths and limitations

Screening used a fixed BMI threshold rather than age- and sex-specific criteria. Applied *post hoc*, the WHO 2007 reference classified 17 participants (56.7%) as having obesity and 13 (43.3%) as having overweight; the CDC 2000 reference classified 15 (50.0%) as having obesity, with one participant falling within the normal range. The sample therefore spans both categories, and the findings should be read as describing adolescent girls with overweight or obesity. Ages were recorded in completed years, so *z*-scores were computed at whole-year boundaries and classification close to a threshold is approximate. Weight status was characterised anthropometrically only; the Edmonton Obesity Staging System for Paediatrics (35) was not applied, although the psychosocial experiences reported here correspond more closely to its mental health and social milieu domains than to BMI category, consistent with evidence that mental health risk in paediatric obesity does not track degree of adiposity (36).

Interviews were conducted in Arabic and quotations were translated by the research team without independent back-translation, so nuance may have been lost. Recruitment was mediated by school principals, and adolescents who were absent from school or unwilling to be screened were not reached. Interviews took place on school premises and, although no staff were present, social desirability may have shaped accounts of both family criticism and personal eating behaviour. Each participant was interviewed once, transcripts were not returned to participants, and member checking was not performed. Coding was carried out by the first author, with the coding frame and theme structure reviewed by the second author; transcripts were not independently double-coded and no inter-coder reliability assessment was undertaken. No socioeconomic or household data were collected, which limits interpretation of the family dynamics described.

The sample was drawn from female adolescents in Riyadh and Arar, which may limit transferability to male adolescents or to other cultural settings, and participants’ descriptions of family interactions are accounts of remembered events rather than direct observations. Strengths include a purposively selected sample of 30 adolescents providing information-rich accounts across the full adolescent age range, a documented and transparent analytic procedure with participant counts reported for every subtheme, and attention to a population and topic that remain under-studied in this setting. As a qualitative exploratory study with purposive sampling, the results are intended to give an in-depth understanding of participants’ experiences rather than to be statistically generalisable.

## 5 Conclusion

Family and community pressure was central to how these female adolescents experienced their weight. Participants described criticism, comparison and monitoring of weight as common, and linked them to eating behaviour, self-esteem, body image, and social participation. Comments focused on appearance were experienced as discouraging. Comments framed around health and delivered supportively were more often described as motivating, particularly where other family members had also lived with overweight or obesity. Interventions for adolescent overweight and obesity should therefore extend beyond dietary and physical activity advice to include reduction of weight stigma, guidance for parents and carers on discussing weight without shaming, and attention to adolescents’ psychological wellbeing. They should also support all adolescent girls in meeting the WHO recommendation of an average of 60 min of moderate-to-vigorous physical activity per day, promoted as a norm for the whole population rather than as a corrective directed at those with higher weight.

## Data Availability

The raw data supporting the conclusions of this article will be made available by the authors, without undue reservation.
