# Supplementary material for: Family and community pressure, obesity-related behaviours, and psychological wellbeing among female adolescents with overweight or obesity: a qualitative study
Source: Front Public Health. 2026 Oct 7;14:1945434. doi: 10.3389/fpubh.2026.1945434 (PMC13683645; doi:10.3389/fpubh.2026.1945434)
Supplement: Supplementary file 1 [file Table_1.docx]

**Supplementary Material 1. COREQ 32-item checklist**

Consolidated criteria for reporting qualitative research (Tong A, Sainsbury P, Craig J. Int J Qual Health Care. 2007;19(6):349–57). Section references are to the revised manuscript.

| **No.** | **Item** | **Response for this manuscript** | **Section** |
| --- | --- | --- | --- |
| **Domain 1: Research team and reflexivity** | | | |
| 1 | Interviewer / facilitator | All interviews were conducted by the first author (AB). | 2.7, 2.10 |
| 2 | Credentials | The first author holds a postgraduate qualification in community health and is an academic staff member at Northern Border University. | 2.10 |
| 3 | Occupation | Academic staff member, Department of Community Health, College of Applied Medical Sciences, Northern Border University. | 2.10 |
| 4 | Gender | Female. | 2.10 |
| 5 | Experience and training | The first author had prior experience in qualitative interviewing and in discussing sensitive topics, including weight and body image, with adolescents. | 2.7, 2.10 |
| 6 | Relationship established | No relationship was established prior to study commencement. The interviewer had no prior relationship with any participant. | 2.10 |
| 7 | Participant knowledge of the interviewer | Participants were told that the interviewer was a female researcher from the university with an interest in adolescent health and family relationships. No further personal information was shared. Participants were also informed of the study purpose, the voluntary nature of participation, and confidentiality. | 2.10, 2.11 |
| 8 | Interviewer characteristics | Reported: gender, occupation, institutional affiliation, prior interviewing experience, absence of prior relationship with participants, and the researcher's prior view that weight-related family comments are often experienced as harmful even when well intended. | 2.10 |
| **Domain 2: Study design — theoretical framework** | | | |
| 9 | Methodological orientation and theory | Qualitative exploratory design with inductive reflexive thematic analysis (Braun and Clarke). The study is not described as phenomenological or as grounded theory. | 2.1, 2.8 |
| **Domain 2: Study design — participant selection** | | | |
| 10 | Sampling | Purposive, criterion-based sampling of female adolescents aged 12–19 years with overweight or obesity who were able to describe their experiences verbally. | 2.3, 2.4 |
| 11 | Method of approach | Recruitment was mediated by school principals in intermediate and secondary schools in Riyadh and Arar, who supported BMI screening of female students. Students identified as having a high BMI for age were invited to interview. | 2.2, 2.4 |
| 12 | Sample size | Thirty female adolescents. | 2.5, 3.1 |
| 13 | Non-participation | Thirty-one eligible adolescents were invited; one declined. No participant withdrew after consenting. The reason for the single refusal was not recorded. | 2.4 |
| **Domain 2: Study design — setting** | | | |
| 14 | Setting of data collection | Private rooms on the premises of the participating schools. | 2.7 |
| 15 | Presence of non-participants | Interviews were conducted individually. No parents or school staff were present. | 2.7 |
| 16 | Description of sample | Thirty female adolescents aged 12–19 years (mean 16.3 years, SD 1.9). Seventeen (56.7%) met WHO 2007 criteria for obesity and 13 (43.3%) for overweight. Mean BMI 31.1 kg/m² (SD 5.0, range 24.8–43.1); mean BMI-for-age z-score 2.27 (SD 0.73, range 1.20–3.66). Participant-level anthropometry is provided in Supplementary Table S1. | 3.1, Table 1 |
| **Domain 2: Study design — data collection** | | | |
| 17 | Interview guide | A semi-structured guide developed from the literature on adolescent obesity, family communication and weight-related pressure, covering eating behaviour, family and social conversations about weight, criticism, social comparison, emotional responses, and coping or support needs. Open-ended questions and follow-up prompts were used. The guide was not formally pilot tested. | 2.6 |
| 18 | Repeat interviews | No repeat interviews were conducted; each participant was interviewed once. | 2.7 |
| 19 | Audio / visual recording | Interviews were audio recorded with participants' consent. No visual recording was made. | 2.7 |
| 20 | Field notes | Field notes and reflexive memos were written after each interview and were read alongside transcripts during familiarisation. | 2.8, 2.10 |
| 21 | Duration | 30–45 minutes. | 2.7 |
| 22 | Data saturation | Sample size was guided by information power rather than a formal saturation assessment. Data collection and analysis proceeded in parallel, and recruitment stopped when the final interviews generated no new codes relevant to the study aim. | 2.5 |
| 23 | Transcripts returned | Transcripts were not returned to participants for comment or correction. This is stated as a limitation. | 4.7 |
| **Domain 3: Analysis and findings — data analysis** | | | |
| 24 | Number of data coders | One. Coding was carried out by the first author. The second author independently reviewed the coding frame and theme structure against the coded data; this was a review of the analytic framework rather than independent double coding of transcripts. No inter-coder reliability assessment was undertaken. | 2.8, 2.9, 4.7 |
| 25 | Description of coding tree | Themes and subthemes, with the number of participants contributing to each, are presented in Table 2. | Table 2 |
| 26 | Derivation of themes | Themes were derived inductively from the interview data. They were not identified in advance. | 2.8 |
| 27 | Software | Coding and data organisation were performed in Microsoft Excel. No dedicated qualitative analysis software was used. | 2.8 |
| 28 | Participant checking | Participants did not provide feedback on the findings. Member checking was not performed and is stated as a limitation. | 4.7 |
| **Domain 3: Analysis and findings — reporting** | | | |
| 29 | Quotations presented | Verbatim quotations are presented for each theme and identified by anonymised participant code (for example P3, P6, P7, P10, P11, P14, P16, P18, P24). Interviews were conducted and coded in Arabic; quotations selected for reporting were translated into English by the research team. The absence of independent back-translation is stated as a limitation. | 2.6, 3.3–3.6, 4.7 |
| 30 | Data and findings consistent | Participant counts are reported for every subtheme in the results and in Table 2, and are drawn from the coding matrix. Frequency terms follow a stated convention (a few = 2–4, some = 5–9, many = 10–19, most = 20–29, all = 30). | 2.8, 3.3–3.6 |
| 31 | Clarity of major themes | Four themes are presented: relationship with food; experiences of criticism; psychological and life-choice implications; and motivation and perceived approaches to weight management. | 3.2–3.6 |
| 32 | Clarity of minor themes | Subthemes are described and illustrated in Table 2, including fast food, healthy eating, dieting, the restraint paradox, sources and nature of criticism, self-consciousness, social withdrawal, self-judgement, marriage prospects, clothing, autonomy, physical activity, and medical intervention. Divergent accounts are reported, including one participant who described no negative weight-related experiences and participants who experienced family communication as supportive. | 3.3–3.6, Table 2 |
